# Radiation Therapy Fields of Dreams: We Built Them—But Did They Come?

**DOI:** 10.1111/1754-9485.70099

**Published:** 2026-04-06

**Authors:** Gerard Adams, Tracey Lee Guan, Elizabeth Anne Burmeister, Bryan Henry Burmeister

**Affiliations:** ^1^ Genesis Care Bundaberg Queensland Australia; ^2^ Rural Clinical School, University of Queensland, Bundaberg Queensland Australia; ^3^ Cancer Alliance Queensland Brisbane Queensland Australia; ^4^ Wide Bay Hospital and Health Service Hervey Bay Australia; ^5^ Genesis Care, Fraser Coast Hervey Bay Queensland Australia

**Keywords:** Cancer Alliance Queensland, radiation therapy, regional and rural, RTU, RTU12

## Abstract

**Introduction:**

Radiation therapy (RT) has been underused in cancer treatment. One possible reason is the long distances some patients must travel for treatment. We performed this study to investigate whether the introduction of RT treatment facilities in regional locations in Queensland influenced RT utility.

**Method:**

The Cancer Alliance Queensland database which links individual patient data to population based and treatment data was used to identify new cancer diagnoses from 2011 to 2020. Cases were grouped according to Local Government Authority (LGA) of residence at the time of diagnosis. Annual use of RT within 12 months of diagnosis (RTU12) for each LGA was calculated. Four regional RT facilities commenced treatment at various points during this time. Variation in year‐to‐year RTU12 for these LGAs was examined before and after opening and compared to comparable figures for Queensland as well as Brisbane, Townsville and Toowoomba (LGAs with long established centres).

**Results:**

Mean RTU12 for Queensland was 24.6%, with minimal year‐to‐year variation. Brisbane, Toowoomba and Townsville LGAs showed stable RTU12 close to the Queensland average. All four regional LGAs without centres in 2010 had lower RTU12 prior to services commencing which improved after installation. For these centres mean RTU12 was 22.1%–95% confidence interval (95% CI) 20.9%–23.3%—before service availability compared to 24.8%–95% CI 24.0% –25.7% (*p* = 0.03)—after service availability.

**Conclusion:**

The installation of regional radiation therapy centers in Queensland appears to be associated with an increase in RT use for patients resident in those LGAs.

**Abbreviations**
CAQ: Cancer Alliance Queensland
LGA: Local Government Authority
QCCAT: Cancer Control Analysis Team
QCR: Queensland Cancer Register
RANZCR: Royal Australian and New Zealand College of radiologists
RR: regional and rural
RT: radiation therapy
RTU: radiation therapy utility
RTU12: radiation therapy utility within 12 months
SACT: systemic anticancer therapy

## 1 Introduction

Radiation therapy (RT) along with surgical oncology and systemic anticancer therapy (SACT) are the three pillars of cancer treatment. Depending on the individual situation, they can be used alone or in combination, with the intent to cure or palliate (live longer and/or live better). While the use of surgical and SACT treatments may be respectively skewed towards the curative and palliative intent ends of the spectrum of treatment goals—RT use is indicated throughout this spectrum and at multiple points on any patient's journey. RT utility (RTU) is defined as the first episode of RT for a new cancer diagnosis and is attributed to the year of diagnosis regardless of the timing of radiation. Estimates from Australia suggest that RT utility (RTU) rates of around 50% would be expected for individual patients when following evidence‐based protocols [1].

Multiple studies have shown historic and longstanding suboptimal RTU within Australia [2, 3] and internationally [4, 5]. Recent reports for Victoria [6] and Queenland [7] show little change in these suboptimal rates over the last decade. Moreover, some reports [3, 6, 8] highlight further inequity with even lower RTU in regional and rural (RR) Australian populations.

A recent publication from the Royal Australian and New Zealand college of radiologists (RANZCR) reports on the increasing number of both RT facilities and the proportion of RT courses delivered in RR locations in Australia during the decade from 2010 to 2020 [9]. This reported showed that Queensland had the largest growth during this period, increasing from 7 to 24 facilities in total. In addition, the recent Cancer Alliance Queensland (CAQ) publication [7] revealed that prior to 2010 the only RT facility outside of the Southeast corner was in Townsville. Much of the growth in facilities during this period did occur in the Southeast, however facilities opened in 5 regional populations (Cairns, Bundaberg, Rockhampton, Fraser Coast and Mackay) at various points during this time meaning that local patients would travel shorter distances for their treatment. However it is unknown if the increased availability of RT in itself results in improvements in RTU in RR communities let alone address the known disparity in outcomes for patients from these communities [10].

For epidemiological analysis, RTU is assigned to the year of cancer diagnosis even though the first RT treatment may be several years later. This is likely to be problematic when trying to detect the impact of specific events such as opening new centres in RR communities. RTU within the first 12 months of diagnosis (RTU12) has been reported as a reasonable predictor of lifetime RTU [11] and will be impacted less by the lag between diagnosis and first RT course, which for some patients can be several years.

The purpose of this study was to use the CAQ dataset to investigate if new RT facilities within RR areas in Queensland did result in increased utility of radiation therapy and it was approved by the radiation oncology subcommittee of CAQ.

## 2 Methods

This retrospective, population‐based cohort study was performed using unit record data from the Queensland Oncology Repository (QOR). Data was obtained in accordance with section 82 of the Hospital and Health Boards Act (2011); therefore, this study is exempt from human research ethics committee approval. QOR collates and matches data from the Queensland Cancer Register (QCR) together with all public and private hospital admissions data, death data and treatment systems including public and private radiation therapy services.

Cancer notification is a statutory requirement throughout Australia, and it is expected that data from QOR covers all people diagnosed with cancer, apart from non‐melanoma skin cancers. People included in this study cohort were Queensland residents newly diagnosed with cancer from 2011 to 2020. Cases were grouped according to Local Government Authority (LGA) of residence at the time of diagnosis. RTU12 for each LGA was calculated for each year as well as for the whole state. In order to allow analysis of RTU12 for at least 2 years before and after local treatment became available, we identified regional LGAs where new centres opened between 2012 and 2018. Four centres became operational during this time (Bundaberg 2013, Fraser Coast 2015, Rockhampton 2016 and Mackay 2018). Variations in year‐to‐year RTU12 for these LGAs were examined before and after opening and compared to comparable figures for the whole state as well as Brisbane, Townsville and Toowoomba (LGAs with long established centres).

For basic patient demographic comparison patients were analysed within two blocks of 5 years (2011–2015 and 2016–2020). This is because the number of patients in some subgroups in each regional new LGA for any given year would be very small. The initial period reflects a time when the regional centres were mostly non‐existent while they were mostly active in the second period.

Descriptive statistics were used to describe RTU12. Mean values with 95% confidence intervals were calculated across the state and with individual LGAs. For the LGAs of interest, mean RTU12 was calculated for the years pre and post installation and compared. A two‐sided *p*‐value of < 0.05 was considered statistically significant.

## 3 Results

Table 1 shows the key characteristics of patients receiving RT in the established LGAs and the new LGAs over the two time blocks. RTU12 was stable or showed small improvements in all demographics in the established LGA. For the new LGAs RTU12 was mostly inferior to the established LGAs in the first time period but showed greater growth in the second time period approaching that of the establishes LGAs.

**TABLE 1:** Key characteristics and RTU12^a^ rates of patients who received radiotherapy within a year of cancer diagnosis by selected LGA groupings, Queensland, 2011–2020.

	New LGAs^b^		Established LGAs^c^	
	2011–2015	2016–2020	2011–2015	2016–2020
	RTU12 rate RT/diagnosed			
Total	23% 3010/13,296	24% 3637/15,175	24% 9046/37,518	25% 10,354/41,568
**Sex**				
Male	20% 1531/7790	21% 1822/8787	20% 4053/20,446	20% 4434/22,402
Female	27% 1479/5506	28% 1815/6388	29% 4993/17,072	31% 5920/19,166
**Age group**				
< 50	27% 420/1570	25% 394/1564	26% 1577/6151	26% 1719/6637
50–59	27% 575/2107	29% 658/2278	28% 1789/6466	28% 1945/6914
60–69	25% 968/3842	25% 1095/4295	27% 2694/10,055	26% 2814/10,724
70–79	22% 805/3596	24% 1087/4459	25% 2076/8342	26% 2701/10,218
80+	11% 242/2181	16% 403/2579	14% 910/6504	17% 1174/7075
**Indigenous status**				
First Nations peoples	25% 65/257	24% 97/404	31% 158/513	30% 201/660
Other Queenslanders	23% 2945/13,039	24% 3540/14,771	24% 8888/37,005	25% 10,153/40,908
**Cancer type**				
Breast	58% 840/1457	62% 977/1586	66% 3207/4832	69% 3731/5440
Lung	42% 487/1164	47% 646/1371	47% 1183/2516	48% 1381/2876
Prostate	20% 447/2292	20% 502/2234	21% 1215/5746	21% 1291/6146
Other	15% 1236/8383	15% 1512/9984	14% 3441/24,424	15% 3951/27,106

^a^ Radiation therapy utility within 12 months of cancer diagnosis (calculated as number of patients receiving radiotherapy divided by the number diagnosed).

^b^ New Local Government Authorities (LGAs): Bundaberg, Fraser Coast, Mackay, Rockhampton.

^c^ Established Local Government Authorities (LGAs): Brisbane, Toowoomba, Townsville.

Table 2 shows the trend in RTU12 for the state of Queensland as well as select LGAs over the 10 years between 2011 and 2020. For Queensland, mean RTU12 was 24.6%–95% confidence interval (95% CI) 24.0%–25.2%. The absolute range was 23%–26% over the period. Three LGAs with established radiation oncology centres within their region for the whole period (Brisbane, Toowoomba and Townsville) all demonstrated a similar stable pattern of RTU12—mean, 24.8%, 23.6%, 24.6%; 95% CI, 24.2%–25.2%, 22.7%–24.5%, 23.8%–25.0%; range 24%–27%, 21%–26% 23%–26% for Brisbane, Toowoomba and Townville respectively.

**TABLE 2:** Annual RTU12^a^ (%) for Queensland state and individual LGA with mean and 95% CI.

Year	2011	2012	2013	2014	2015	2016	2017	2018	2019	2020	Mean over 10 years	95% CI
Queensland	25	24	23	24	24	24	25	25	26	26	24.6	24.0–25.2
Brisbane	25	24	24	25	24	25	24	25	25	27	24.8	24.2–25.4
Toowoomba	24	21	22	23	24	24	25	26	23	24	23.6	22.7–24.5
Townsville	24	26	23	24	25	25	24	23	26	26	24.6	23.8–25.3
Bundaberg	22	21	23	28	26	26	27	25	27	28	25.3	23.8–26.9
Fraser Coast	20	18	23	23	25	25	22	25	23	23	22.7	21.3–24.1
Mackay	25	29	21	22	21	16	21	25	22	24	22.6	20.5–24.7
Rockhampton	22	19	19	24	25	22	25	23	23	25	22.7	21.3–24.1

*Note:* For those LGAs where the first radiation therapy treatment facility became operational during this time period the years prior to service availability are in blue and the years with service availability are in green.

Abbreviations: 95% CI, 95% confidence interval; LGA, Local Government Authority.

^a^ Radiation therapy utility within 12 months of cancer diagnosis (calculated as number of patients receiving radiotherapy divided by the number diagnosed).

For the 4 LGAs (Bundaberg, Fraser Coast, Mackay and Rockhampton) with new centres, Table 2 illustrates the spread of RTU12 over the period with the years before opening shaded blue and the years post installation in green.

Table 3 demonstrates the difference in RTU12 for these LGAs on opening new centres. Yearly mean RTU12 improved in all 4 centres post installation—Bundaberg 26.7% vs. 22.0%, Fraser Coast 23.6% vs. 21.8%, Mackay 23.0% vs. 22.5% and Rockhampton 24.0% vs. 21.8%.

**TABLE 3:** Mean RTU12^a^ (%) for the four LGA and in combination for the years before and after service availability.

	Before installation		After installation		*p*
	Mean	95% confidence interval	Mean	95% confidence interval	
Bundaberg	22.0	20.9–23.3	26.7	25.9–27.7	
Fraser Coast	21.8	19.4–24.2	23.6	22.4–24.8	
Mackay	22.5	19.8–25.2	23.0	21.0–25.0	
Rockhampton	21.8	19.8–23.8	24.0	22.7–25.1	
Combined	22.1	20.9–23.3	24.8	24.0–25.7	0.03

*Note:* Two‐sided *t*‐test.

Abbreviation: LGA, Local Government Authority.

^a^ Radiation therapy utility within 12 months of cancer diagnosis (calculated as number of patients receiving radiotherapy divided by the number diagnosed).

When all four LGAs were analysed together the RTU12 improved to 24.8% (95% CI 24.0%–25.7%) from 22.1% (95% CI 20.9%–23.3%) *p* = 0.03 with the opening of a local service.

## 4 Discussion

The analysis presented here clearly demonstrates an improvement in RTU12 in the four LGAs where new radiation therapy centres were installed during the time period of the study. In comparison, the state as a whole and those LGAs with long established facilities showed a relatively flat and consistent pattern over the same period. Moreover, the clear gap in RTU12 in underserved locations compared to the state average and well serviced locations is almost eliminated with the introduction of local RT services.

While previous studies have shown that regional radiotherapy centres have improved access [12], to our knowledge this is the first Australian paper using individual patient data (incidence and RT utility) from the specific regional locations during this time period to demonstrate an increasing utility directly attributable to the opening of individual regional facilities.

Butler [13] did report more patients accessing radiation therapy when a facility opened in Orange—however the denominator for the calculation was estimated population cancer incidence from statewide data and so may not accurately reflect changes in RTU. In addition, Merie et al. [14] did report an improvement in RT use in New South Wales within a year of cancer diagnosis from 22% in 2004–2006 to 25% in 2009–2011 noting that accessibility improvements for regional patients likely contributed with more patients (82% vs. 78%) living < 50 km from an RT facility. However, the focus of this paper was not changes in utility over time following the opening of a certain regional facility.

Utility of radiation within 1 year of a cancer diagnosis will vary between tumour types; nevertheless, long‐term data from the Ontario Cancer Registry [11] suggests that RTU12 is a reasonable predictor of RT use within 20 years of a cancer diagnosis (and hence lifetime). RTU is estimated to be 1.3 times RTU12 in a general population of cancer patients. Assuming this is true, for these Queensland centres, a 2.7% uplift in RTU12 represents a potential eventual improvement in RTU of 3.5% by investing in local centres. Given that the RTU as a state was flat at 30% [7] during this period (and even less for these regions)—this potentially results in a greater than 10% relative increase in RTU in these regions.

Given that RTU represents only the initial RT treatment for a particular cancer treatment and retreatment rates during the period of this study were around 25% [15], the impact on the total number of patients from these regions receiving RT is likely to be very meaningful as it can be assumed more patients will also receive second and subsequent courses if they have less distance to travel.

While the Queensland data showed a stable RTU12 over the 10‐year period, there were some demographics such as older patients and breast cancer that showed small improvements during the second part of the decade (Table 1). Given that the new centres in the regional LGAs opened at different points during that decade, analysing the annual RTU12 for each subgroup before and after opening of the new centres would be problematic. Nevertheless, when the decade is split into two time periods, 2011–2015 and 2016–2020, the data do show that the improvement in RTU12 in the second period (when the new regional centres were mostly operational) was greater for older patients, as well as patients with breast and lung cancer—and that these increases were greater than those seen at established centres.

There are limitations to this study. While these data clearly do demonstrate an uplift in RTU, they cannot be used to comment upon the appropriateness of case selection or the quality of RT delivered. However, given the well documented consistent and longstanding suboptimal utility of RT, overuse is much less of a risk than underuse.

Another observation is that, for these four regions, those with more active years during the time course of the study (seven for Bundaberg and only two for Mackay) showed a greater rise in mean RTU12 (4.7% vs. 0.5%). This suggests that it may take time to impact change (e.g., alter established referral patterns) resulting in an underestimate of local impact of a new centre in the early years.

For individuals making the choice between treatment options (e.g., surgery or RT for prostate cancer or early lung cancer) the availability of RT in their hometown versus the requirement to travel for the surgical option could be the deciding factor in their choice. While services in the four towns are different, the options for local specialist cancer surgery are more limited than in the major cities. This is in keeping with the observation in Table 1 of the relatively large increase in RTU12 in lung cancer in the regional LGAs. Given the fact that many patients with prostate cancer do not have an intervention in the initial year of diagnosis, change in RTU12 will not be the best tool to evaluate RT use in prostate cancer. The local availability of radiotherapy services as opposed to the need to travel for the surgical alternatives could partly explain the observation that RTU12 in Bundaberg exceeded the state average once the centre opened.

It should be noted that these figures reflect RT use in patients from the local LGA and not activity in the RT facility. It is likely (and indeed appropriate) that some patients will still travel to metropolitan centres for specialised techniques that might not be available locally. The dynamics of this relationship are beyond the scope of this study.

Another potential limitation is that patients are attributed to the LGA of residence at the time of cancer diagnosis. At the point of treatment some will have moved residence to other LGAs. This is a phenomenon that will affect all patients in all LGAs. In addition, limiting the study to 1 year after cancer diagnosis will minimise any effect. Moreover, if there is an overall effect of change of residential address on RTU data, it is likely that patients will move from resource‐poor regional areas to resource‐rich metropolitan areas. Therefore, it is unlikely this limitation would have a negative impact on the interpretation of this study.

It is also important to remember that although these locations are regional—they are all relatively large regional towns and cities with catchment areas mostly in excess of 100,000 people. It is not known whether centres in smaller populations would be as successful in improving RTU, given the many known challenges in establishing and resourcing RT facilities in regional areas within Australia [16].

It is important to remember that the stagnant RTU12 for the regions with established centres during this time does not mean that there was no increase in demand for RT services in established centres when the new centres opened. Table 1 shows that for the established LGAs there was an increase in total RT cases from 37,518 to 41,568 (10.5%) from the 2010–2015 to the 2016–2020 period. With growing cancer incidence due to growing and ageing populations, more courses are required each year to maintain current utility.

Additionally, while the improved RTU12 in the new centres is a positive finding, the utility of RT in Queensland, including these regions, is still suboptimal. The reasons for this chronic under utility are manifold and complex. This study suggests that travel distance is one. Resourcing is another. The new centres did supply extra resources for the state as a whole but not enough to maintain statewide RTU let alone close the gap on optimal RTU. While increasing the number and improving the distribution of resources is important, more work needs to be done to reduce the persistent gap between actual and optimal RTU.

This article confirms yet again the historic under utility of RT in regional areas within Australia. With patient specific data on cancer incidence and RT utility in individual LGAs this paper confirms that placing RT facilities within regional communities can make meaningful differences in the utility of RT in those populations. While the opening of these centres made an important impact in their own communities by improving RT utility, it should be noted that this only increased RTU12 towards the state level which is still far below the optimum rate. Many regional patients live in towns smaller than these. It is unlikely that patients in smaller regional and rural communities will procure RT centres in their communities. So while it is good news that RT utility can be improved in this way, the provision of regional RT centres alone will not be enough to negate the impact of the tyranny of distance in cancer care.

## Author Contributions

All authors contributed to the writing and review of the manuscript and accept responsibility for the final version.

## Conflicts of Interest

The authors declare no conflicts of interest.

## Data Availability

The datasets generated during and/or analysed during the current study are not publicly available due to confidentiality restrictions but may be available from the corresponding author upon reasonable request.
